# Strain-Enhanced Large-Area Monolayer MoS_2_ Photodetectors

**DOI:** 10.1021/acsami.4c00458

**Published:** 2024-03-19

**Authors:** Borna Radatović, Onur Çakıroğlu, Valentino Jadriško, Riccardo Frisenda, Ana Senkić, Nataša Vujičić, Marko Kralj, Marin Petrović, Andres Castellanos-Gomez

**Affiliations:** † Center for Advanced Laser Techniques, Institute of Physics, Bijenička 46, 10000 Zagreb, Croatia; ‡ Materials Science Factory, Instituto de Ciencia de Materiales de Madrid (ICMM-CSIC), 28049 Madrid, Spain; § Physics Department, Politecnico di Milano, 20133 Milan, Italy; ∥ Physics Department, Sapienza University of Rome, 00185 Rome, Italy

**Keywords:** MoS_2_, strain, strain sensor, photodetector, atomic force microscopy, PL spectroscopy, photocurrent spectroscopy

## Abstract

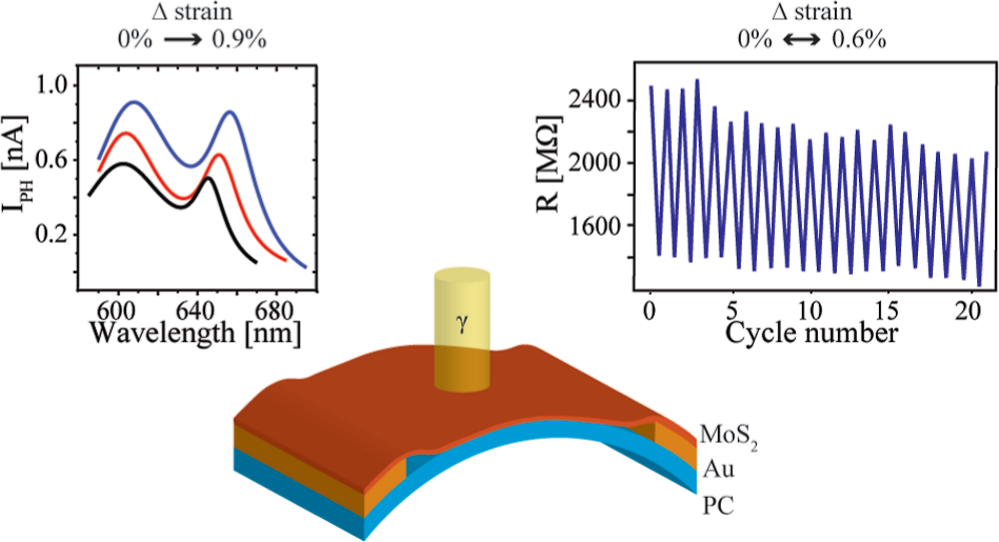

In this study, we show a direct correlation between the applied mechanical strain and an increase in monolayer MoS_2_ photoresponsivity. This shows that tensile strain can improve the efficiency of monolayer MoS_2_ photodetectors. The observed high photocurrent and extended response time in our devices are indicative that devices are predominantly governed by photogating mechanisms, which become more prominent with applied tensile strain. Furthermore, we have demonstrated that a nonencapsulated MoS_2_ monolayer can be used in strain-based devices for many cycles and extensive periods of time, showing endurance under ambient conditions without loss of functionality. Such robustness emphasizes the potential of MoS_2_ for further functionalization and utilization of different flexible sensors.

## 1 Introduction

Two-dimensional (2D) materials exhibit impressive resilience to mechanical deformation, which, in conjunction with the tunability of their properties with strain, offers significant advancements for the next generation of straintronics.^1−3^ Generally, strain in 2D materials introduces lattice deformations such as changes in the bond lengths and angles.^4,5^ In addition to crystal lattice modifications, strain in semiconducting 2D materials modifies their bandgap, which causes various alterations, e.g., the shift of exciton energies or change of resistance, usually quantified by gauge factors (GFs).^6^ For instance, tensile strain fields can influence exciton energy and spectral redshift due to bandgap reduction, but funneling effects can also be observed where photoexcited excitons concentrate toward higher strain regions.^4,7,8^ For all 2D materials, strain induces a change in the electrical resistivity, causing a piezoresistive effect that is most pronounced for semiconducting materials.^8,9^ With the applied strain, the energy of the valence (conduction) band increases (decreases), respectively, resulting in reduced bandgap and enhanced conductivity, which can be quantified by the piezoresistive GF (GF_P_).^7,8^ Furthermore, for 2D materials with an odd number of layers, where the inversion symmetry is broken, strain induces charge accumulation on the material’s edges and a change of electrical potential, which results in a piezoelectric effect.^10^ Additionally, the change of electronic structure and the bandgap shrinkage cause a reduction of intervalley scattering and effective mass lowering, resulting in strain-enhanced MoS_2_ field-effect transistor mobility.^11−13^ Moreover, it was shown that a strain-engineered MoS_2_ monolayer could capture a broad range of the solar spectrum and spatially concentrate excitons or charge carriers as the optical bandgap also continuously changes with the applied strain.^14^

Optoelectronic devices based on 2D materials offer an efficient platform for converting photons into electric signals, primarily due to their large surface area and broadband photodetection.^15^ For instance, graphene efficiently absorbs light over a broad range of wavelengths, making it suitable for photodetectors from UV to THz.^16,17^ However, graphene does not have a bandgap, which confines its ability to absorb light in the visible spectrum. This can be a limitation in specific photodetector applications, especially when high sensitivity in the visible range is required. In contrast to graphene, transition-metal dichalcogenides (TMDs), such as MoS_2_ and WS_2_, have a finite bandgap combined with the large electronic density of states, resulting in high optical absorption and ultrafast charge transfer, which makes them more suitable for photodetectors in the visible and near-infrared spectrum.^18,19^

The majority of previous papers on 2D materials for straintronic and flexible optoelectronic devices are based on exfoliated monolayers and chemical vapor deposition (CVD)-grown multilayers.^7,20−22^ Although multilayer 2D materials are in principle easier to handle and are mechanically more stable in comparison to monolayers, the MoS_2_ monolayer has a higher light absorption in energies lower than the A exciton and overall better light emission than that of its multilayer counterparts.^23^ While exfoliation offers a simple method for obtaining monolayers, it has a setback in the view of integration with current standards and processes exploited in the semiconductor industry; also, the samples are mostly limited in lateral sizes to micrometer dimensions.^22,24,25^ Notably, previously reported large-scale MoS_2_ monolayer optoelectronic devices were usually coated with different polymers or other materials, which prevented options for further functionalizations.^26^

Devices investigated in this work were based on a CVD-grown MoS_2_ monolayer sheet with more than 1 cm lateral dimensions. After the initial characterization of optical and morphological properties, we have demonstrated that large-scale MoS_2_ monolayers can be repetitively and continuously used as strain sensors and flexible photodetectors. In particular, by performing photocurrent spectroscopy under the applied uniaxial tensile strain (ε) up to 0.9%, as confirmed by the determined A and B exciton GFs (GF_A_ and GF_B_) that agree with previous reports, we have demonstrated that ε causes the increase of the MoS_2_ monolayer photoresponsivity several times.^5,6,27^ The exhibited overall large photocurrent and long response time of the investigated devices are characteristics of photodetectors dominated by photogating mechanisms.^28^ In addition, the reported simultaneous increase in response time at higher amounts of strain indicates that photocurrent enhancement is probably caused by the change of trap states or hole trapping time.

## 2 Experimental Methods

### 2.1 Differential Reflectance Spectroscopy

A Motic BA310 metallurgical microscope equipped with a 50× objective (NA = 0.55 and WD = 8.2 mm) was modified to enable optical inspection with a microscope and spectroscopy analysis of the same sample area. A halogen light source was used as an optical probe that was split at a 50:50 ratio by a modified trinocular. 50% was coupled to an optical fiber and used for signal analysis by the Thorlabs ccs 200/m CCD spectrometer, while the other 50% was used for sample imaging.

### 2.2 Atomic Force Microscopy

Atomic force microscopy (AFM) images were recorded with a JPK Nanowizard Ultra Speed AFM instrument under ambient conditions. Noncontact AC (tapping) mode was used for data acquisition with a set point of around ∼65%. Bruker TESP-V2 silicon tips with a nominal spring constant of 37 N/m, a tip radius of 7 nm, and a resonant frequency of 320 kHz were used. Images were processed with JPK Data Processing software and WSxM software.^29^

### 2.3 Photoluminescence Mapping

For the acquisition of the MoS_2_ photoluminescence (PL) spectra map, the excitation laser energy was 532 nm and the laser power on the sample was 250 μW. The incident laser was focused by a 50× infinity corrected objective (NA = 0.75) and excited a 0.68 μm diameter spot on the sample (determined from the full width at half-maximum of the beam excitation profile at the sample). The PL signals were collected through the same microscope objective, filtered by a set of Bragg filters, coupled in a 50 μm core fiber that serves as a confocal detection pinhole, and analyzed in a 50 cm long spectrometer (Andor Shamrock 500i-B1) in combination with a 150 l/mm diffraction grating and cooled EM CCD detector (Andor Newton 971). Part of the setup was the XY stage, on which the sample was mounted for optical characterization. The setup was automated so that the stage moved line by line in 500 nm steps in the *x*-direction and 700 nm in the *y*-direction, while the spectrum was acquired at each step. The optical PL map was measured in air and at room temperature.

### 2.4 *I*–*V* Characterization

Electrodes fabricated by shadow mask evaporation, with masks purchased from Ossila, were connected to the source measure unit (SMU) with standard laboratory wires by dropping silver paste on the pads of the electrodes. *I*–*V* characterization was performed by measuring the current between the source and drain electrodes while sweeping the bias voltage applied to the electrodes. Keithley 2450 SMU was used for all transport measurements.

### 2.5 Photocurrent Spectroscopy

Photocurrent spectroscopy was performed by measuring the photocurrent during illumination by light with different wavelengths.^22,30^ Two setups were used; the first one uses LED lights with specified wavelengths (365, 405, 420, 455, 470, 505, 530, 565, 595, 617, 625, 660, 780, and 850 nm) and a power of *P* = 1.25 μW with a light spot of 400 μm diameter. The second setup used a Bentham TLS120Xe light source that can be continuously set to different wavelengths in arbitrary steps. For a closer inspection of the exciton energy range, the second setup was used with wavelengths from 580 to 680 nm in steps of 5 nm.

## 3 Results and Discussion

### 3.1 MoS_2_ Homogeneity and Device Morphology

**Figure 1:**
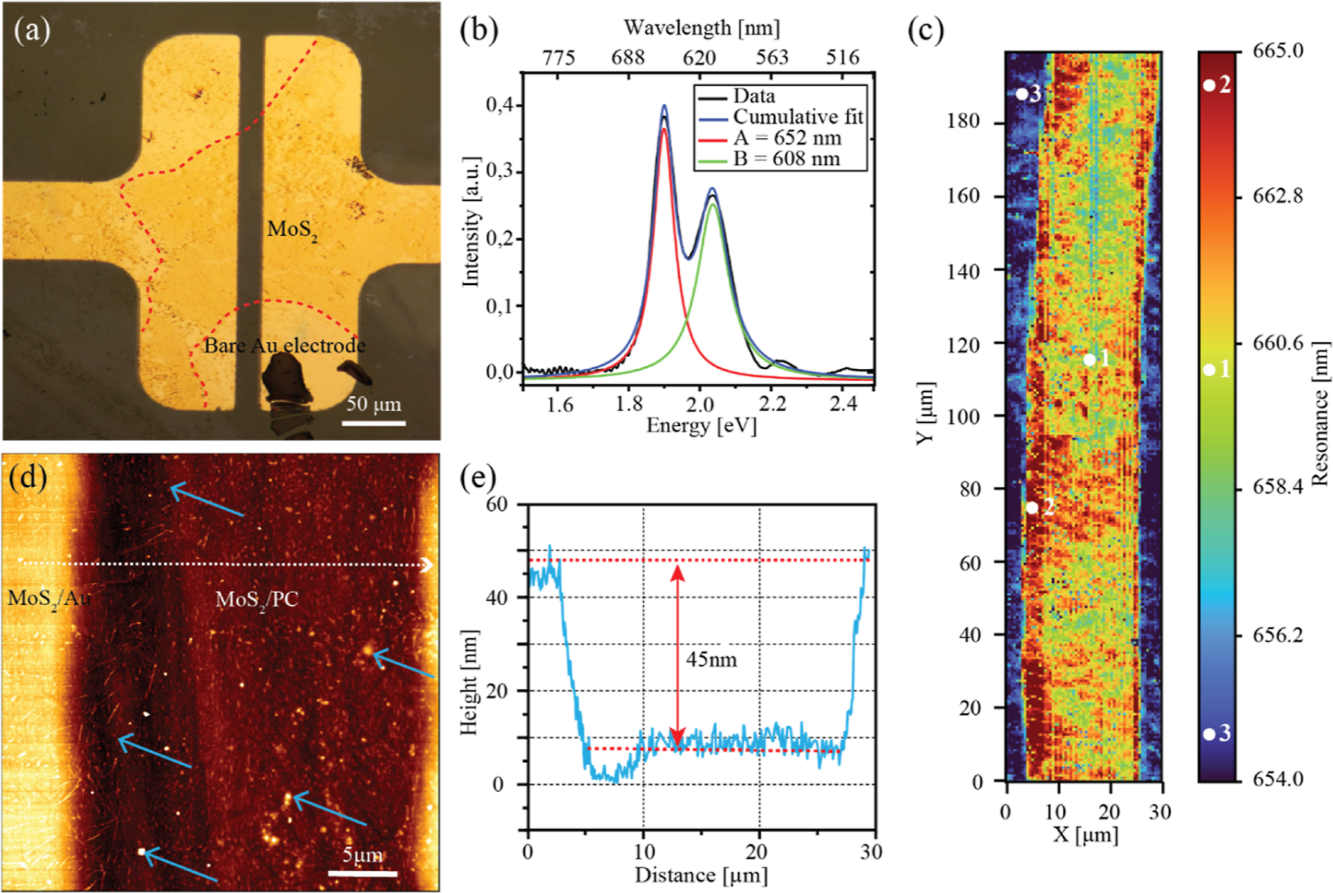
Uniformity characterization in the device channel. (a) Optical micrograph of the MoS_2_ monolayer transferred on top of the PC sheet with a prefabricated pair of Au electrodes. The MoS_2_ area over electrodes is indicated with a dashed red lines. (b) Microreflectance spectrum of the MoS_2_ monolayer in the channel. Exciton A and B Lorentzian fits and the cumulative fit are indicated with red, green, and blue lines, respectively. (c) PL map of the A exciton resonance of the device channel. Values taken as characteristic A exciton energies for MoS_2_ in the channel at the PC, MoS_2_ on the edge of the electrode, and MoS_2_ at Au electrodes are indicated with 1, 2, and 3, respectively. (d) AFM topographic image of the sample with no visible cracks in a 30 μm × 30 μm sized region. The blue arrows indicate transfer-induced contaminations. (e) Channel line profile taken along the white dashed arrow in (d).

We investigated a large-scale MoS_2_ monolayer sheet synthesized by CVD growth on a Si/SiO_2_ wafer. Such samples require transfer from a rigid substrate to a flexible one to enable strain engineering by bending or stretching the substrate. We have used the polydimethylsiloxane (PDMS) stamping method to transfer MoS_2_ monolayers on top of the polycarbonate (PC) sheet with prepatterned Ti/Au electrodes via shadow mask lithography.^21,22,31^ Each step of the transfer method is described in detail in the Supporting Information; see Figure S1. The first confirmation of a successful transfer came from a quick inspection with an optical microscope and reflectance spectroscopy, as has been done previously for different TMDs.^32^ Since our samples had centimeter lateral dimensions, some areas of MoS_2_ contained multilayers that occurred in the middle of the nucleation points or at intersecting grain boundaries of MoS_2_. Optical microscopy can quickly confirm that the uniformly clean MoS_2_ sheet is placed in the adequate position over the electrodes, as shown in Figure 1a, where a deliberately partially transferred sheet is depicted to indicate the color contrast difference between the uncovered parts of Au electrodes and the MoS_2_/Au area. It is important to note that all measurements were performed with MoS_2_ sheets spanning across the device channel. In parallel with the optical inspection, we performed differential reflectance spectroscopy in multiple spots in the channel to confirm that the transferred MoS_2_ sheet is a monolayer. The reflectance spectrum in Figure 1b shows characteristic measured data and resultant Lorentzian fits for the A (652 nm ≈ 1.90 eV) and B (608 nm ≈ 2.04 eV) exciton energies in correspondence with previous reports of monolayer MoS_2_.^33,34^

We measured PL maps to confirm that the whole device channel is covered with MoS_2_, as shown in Figure 1c, where the positions of A exciton energies are depicted for the large part of the channel. The unstrained energy value of the A exciton peak was averaged over multiple positions in the middle of the channel to *E̅*_A_ = 659 nm ≈ 1.88 eV to determine the exact amount of strain on the surface. Alterations in the energy of the exciton in MoS_2_ monolayers depending on the type of optical spectroscopy (absorption vs emission), i.e., Stokes shift, were expected as the PL corresponds to the distribution of the lowest exciton energy states, while the micro reflectance spectrum represents the distribution of all exciton states.^35−37^ As seen from the PL map, the channel looks relatively uniform; however, certain deviations in A exciton energy can be noticed, which could be caused due to the local strain variation, the presence of defect states intrinsic to CVD-grown materials, multilayer contribution, or different substrates and MoS_2_.^38^ For example, the blueshift of 5 meV for the exciton energy values of MoS_2_ at Au electrodes (position 3) in Figure 1c with respect to the MoS_2_ at the PC in the channel (position 1) can be attributed to a change in dielectric screening and/or doping arising from two different substrates.^39,40^ In correspondence with the previously mentioned variations, the strain caused by bending the monolayer (position 2) causes a redshift of the A exciton energy by −18 ± 6 meV, which would correspond to less than 0.4% of the strain when calculated from the obtained GF_A_ and GF_B_ values. The redshift caused by local bending on the edges gradually reduces toward the middle of the channel (position 1). Additionally, certain deviations in PL emission at the energies of A exciton in the middle of the channel are probably due to different local strains (variations up to 0.2%) caused by the substrate morphology, probably governed by contaminations between PC and MoS_2_.

Sample morphology in the channel was investigated via AFM to determine whether the energy variation originated from the multilayer contribution or local strain inhomogeneity. As shown in Figure 1d, the majority of the channel is covered by a homogeneous MoS_2_ monolayer with few contaminations (most likely PDMS residues and contaminations from the transfer). Additional morphology inhomogeneity originates from cracks, ripples, and holes in the monolayer sheet, as depicted in Figure S2a,b. Although all of these features can cause local strain and are a source of structural impairment, most of the channel area contains an adequately transferred MoS_2_ sheet whose height follows the Au–PC–Au profile with bending at electrode edges, as shown in the line profile in Figure 1e.

### 3.2 Strain Tunability of MoS_2_ Resistance

**Figure 2:**
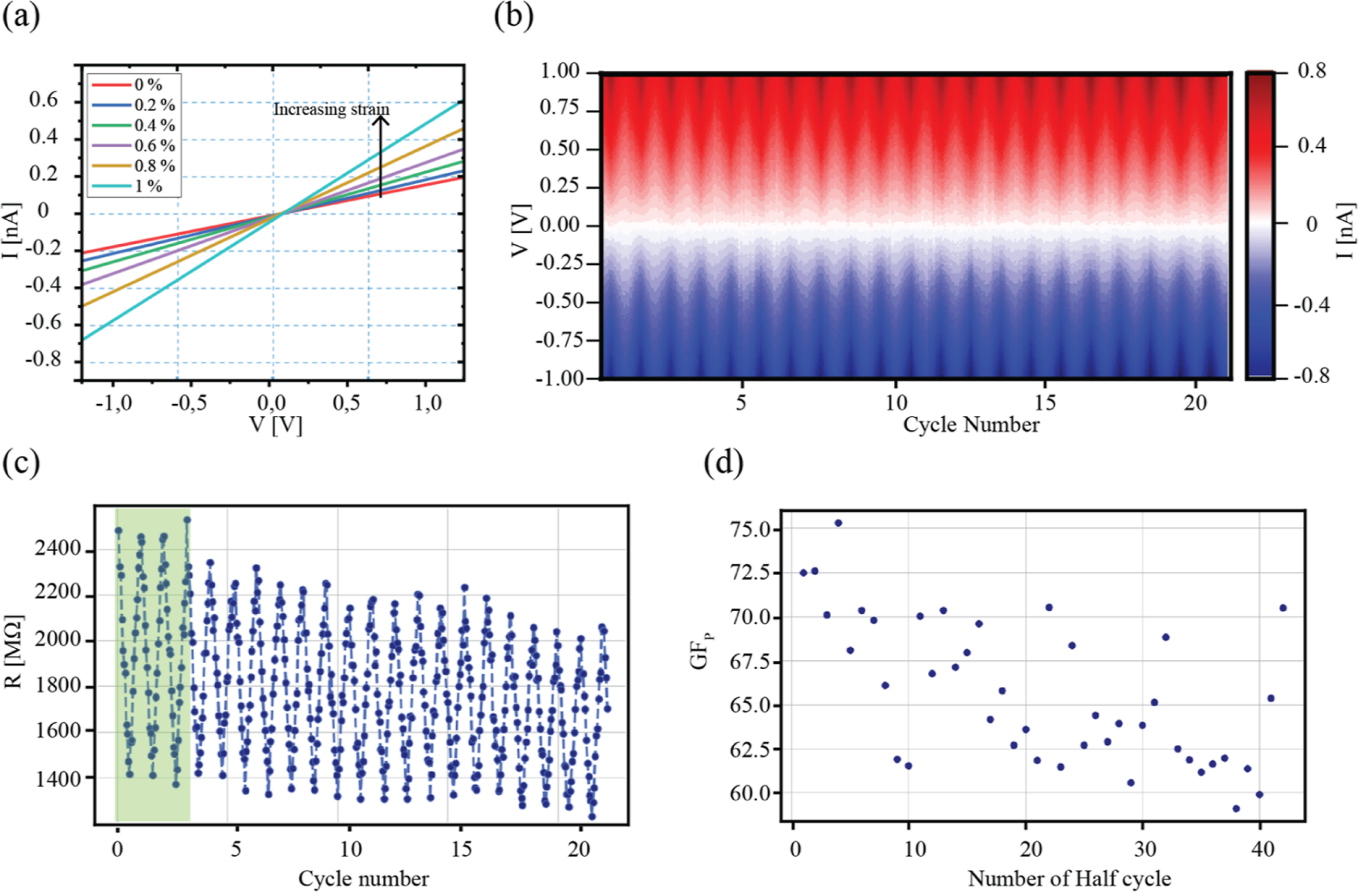
Piezoresistive effect in the MoS_2_ monolayer. (a) *I*–*V* characterization under different levels of applied ε up to 1%. (b) Evolution of current versus voltage during 20 bending cycles, where each cycle consists of 21 steps in which strain increases from 0% up to 0.6% and back to 0%, all in 0.06% increments, i.e., decrements. The applied voltage is shown on the *Y*-axis, a cycle number on the *X*-axis, and a measured current as a color scale. The red/blue color indicates the current measured under positive/negative bias, respectively. Darker shades of red and blue indicate the highest current (lowest resistance, under 0.6% of applied ε), while brighter corresponds to the lowest current (highest resistance, under 0% of applied ε). (c) Resistance vs cycle number, with an indicated green rectangle that shows the first three cycles after which prestrain is partially released. The maximum resistance in each cycle corresponds to 0% of applied ε while minimum to 0.6%. (d) GF_P_ for each new half cycle over the 20 subsequent cycles of bending.

After the device was fabricated and preliminary characterized, its current vs voltage (*I*–*V*) characteristics were measured under applied ε to determine GF_P_. First, *I*–*V* measurements were performed, measuring the current between the source and drain electrodes while sweeping the bias voltage. This process was repeated for different amounts of uniaxial ε induced by the three-point bending motorized stage, as described in the work by Çakıroğlu et al.^21^ The GF_P_ value can be determined by measuring *I*–*V* sweeps under different amounts of strain, as GF_P_ = (Δ*R*/*R*_0_)/ε, where *R*_0_ is the resistance without applied strain and Δ*R* is the change of the resistance under applied strain.^8^Figure 2a shows *I*–*V* sweeps under different amounts of applied strain, up to a maximum value of 1%. By increasing ε in MoS_2_, resistance decreases. The calculated average GF_P_ value is GF_P_ = 70 ± 3, roughly 35% of the previously reported highest value for MoS_2_ monolayers.^8,13,21,22^ It is important to note that previous reports on monolayers were mostly for exfoliated flakes, while on large-scale CVD-grown 2D materials, multilayer regions can occur in addition to local rip folding during the transfer. Although previous reports show that multilayers, such as trilayers, result in lower GF_P_ values,^7,8^ here, as previously discussed, reflectance and PL spectra showed that A and B exciton positions correspond to the MoS_2_ monolayer over the majority of the channel. So, most likely, transfer-induced defects such as contaminations and cracks disclosed in AFM topographic images in Figures 1d and S2, respectively, give rise to a reduction of our GF_P_ value. So far, the highest GF_P_ values for the CVD-grown MoS_2_ monolayer were reported by Datye et al. with values up to 200.^13^ However, their channel length was around 8 μm, approximately three times shorter than in this work, thus exhibiting reduced crack and contamination effects.

After device characterization with up to 1% strain applied, the samples were subsequently tested over a time period longer than 60 h, which contained over 20 bending cycles up to 0.6% strain. Each cycle consisted of 10 equal load steps and the subsequent 10 unload steps. *I*–*V* sweeps were performed at the start of the load–unload cycle and after each step. As a result, a total of 21 *I*–*V* measurements correspond to one complete cycle, with the first and 21st measurements at 0% of ε and the 11th measurement at the highest ε level in the cycle. Figure 2b shows the corresponding *I*–*V* maps, where the *Y*-axis corresponds to the voltage applied, the color scale indicates the measured current, and the *X*-axis is set as the cycle number. Our results suggest that even a non-encapsulated MoS_2_ monolayer can be used in strain-based devices for many cycles, showing strain-independent endurance under ambient conditions without losing functionality.

Figure 2c shows the dependence of the resistance on step number for more than 20 cycles. From there, it can be seen that the resistance decreases with the strain increase (load) and subsequently rises to the starting value when strain is released (unload) after a complete cycle. For the first three cycles, the resistance measurements are essentially repetitive. However, in the fourth cycle, a drop in resistance of about 10% happens due to the prestrain release, which was located around bubbles, and a mismatch of MoS_2_ sheet around cracks that most likely occurred after the transfer of MoS_2_.^26,41^ After that, resistance continues to drop gradually by an overall additional ∼10% for the remaining cycles. This decrease is attributed to the exposure to the environment in which various molecules from the air can bond to the surface of 2D materials over time.^42^ The stability of the fabricated device can be confirmed by observing GF_P_ during bending cycles. Figure 2d shows GF_P_ for each half-cycle, where an odd number of half-cycles corresponds to applying ε by bending and an even number of half-cycles corresponds to the release of strain to 0%. Even though GF_P_ changes for each new cycle, variations after the first three cycles are less than ±10% from an average GF_P_ = 66 ± 5, which signifies along lifetime of the fabricated device confirmed during a few days of operating hours under ambient conditions. Such results endorse the application of large-scale monolayers of MoS_2_ as highly precise and stable strain sensors or tactile sensors.

### 3.3 Strain Enhancement of MoS_2_ Photodetectors

**Figure 3:**
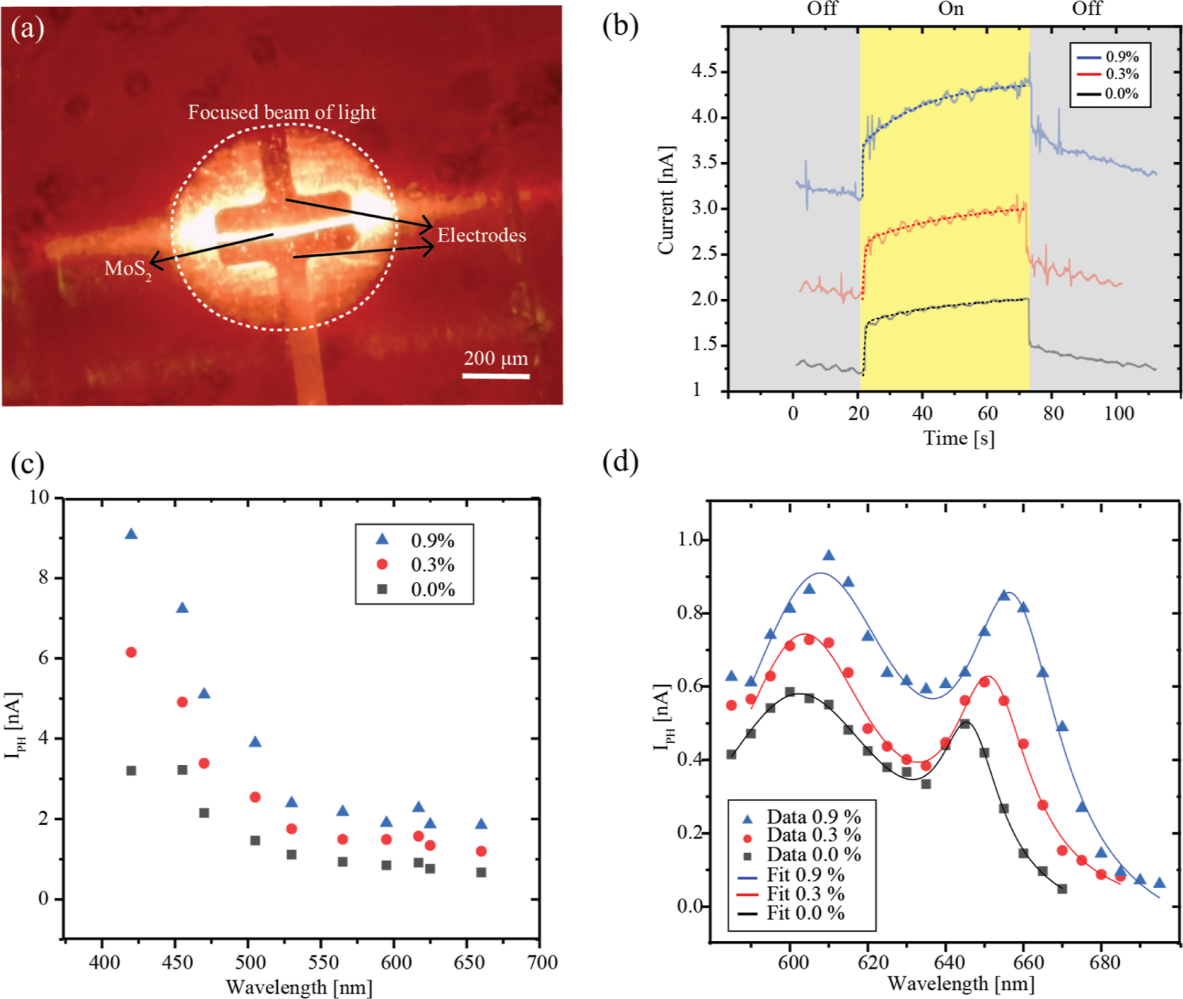
Strain-enhanced MoS_2_ photoresponse. (a) Optical microscopy image of the device under exposure to light. (b) Photoresponse of MoS_2_ at a light illumination of 645 nm, with the light turned on for 45 s and switched off afterward. Measured current and corresponding double exponential fits are shown for 0.0, 0.3, and 0.9% of tensile strain. (c) Photocurrent spectroscopy of the MoS_2_ monolayer sheet by exposure to the LED light with different wavelengths at 0.0, 0.03, and 0.09% of tensile strain. (d) Higher-resolution photocurrent spectroscopy of the MoS_2_ monolayer sheet with exposure to a continuous light source with different wavelengths at 0.0, 0.3, and 0.9% of tensile strain. Data show the energy range in which B (604 nm) and A (645 nm) exciton peaks are found and fitted with two Lorentzians.

After confirmation of the adequate response of MoS_2_ devices under strain, they were further characterized by photocurrent spectroscopy at various amounts of ε. The photocurrent *I*_ph_ was determined by measuring the current before, during, and after illumination by monochromatic light, while a potential of 1 V was applied to MoS_2_ via electrodes, as shown in Figure 3a. Two types of light sources were used for different spectral ranges and wavelengths: the first setup, which uses LED lights with specified wavelengths, and the second light source, which can be set to different wavelengths in arbitrary steps (see Methods for more details). Figure 3b shows the OFF–ON–OFF illumination period with a 645 nm light at different strain levels. After illumination of the sample, rapid current growth can be seen, with a slow saturation trend. Rapid response is also seen when illumination is turned off and the decay starts. Due to cyclic illumination and a long saturation time, the decay from the previous illumination is still present seconds before the new illumination period, as visible in Figure 3b. Device response time can be derived from the 10 to 90% method, i.e., as the time which passes from achieving a 10 to 90% value of the saturation photocurrent.^43,44^ Other methods of fitting and analysis were also used, but we did not achieve better fits with other functions, so the most used method in the field was appropriate for our data.^45^ Additionally, by subtracting the off-current (*I*_OFF_ = 10% *I*_saturation_) from the on-current (*I*_ON_ = 90% *I*_saturation_), the persistent photoconductivity effect is eliminated from further photocurrent analysis.^45^

Two mechanisms control photocurrent generation: photoconductance and photogating.^28^ Through the response time of the device, it can be determined which mechanism is dominant. On one hand, the typical response time for photoconductance-dominated devices is less than 10 ms, in which case photoexcited electrons and holes are separated by the bias voltage, which leads to an increase in the current in the material. On the other hand, the response time for photogating-dominated devices can be more than 10 s, in which case photoexcited electrons drift due to the bias voltage while holes get trapped, and as a result, extra electrons move to the channel. After each drifting electron reaches the drain electrode, one new electron must jump from the source electrode due to charge conservation in the channel. In that way, the photoconductive gain is proportional to the ratio between the electron drifting time and the hole trapping time.^22^ Generally, photoconductive-dominated devices have a fast response but low photoresponsivity values, while photogating-dominated ones are usually slower but achieve higher values of photoresponsivity.^46^ Our devices show a slow response time from ∼4 s at 0.0% to ∼7 s at 0.3% and ∼8 s at 0.9% strain, indicating that devices are dominated by a photogating mechanism that becomes more pronounced with the application of strain. Although here we did not perform power dependence measurement of the photocurrent, in our previous papers,^21,22^ sublinear power dependence of photocurrent accompanied by a long response time was presented.

Figure 3c shows the generated *I*_ph_ by illuminating the channel with the LED light source at 0.0, 0.3, and 0.9% of ε. With the increase of ε, a notable *I*_ph_ increase and a redshift of exciton energies are visible. Consequently, photoresponsivity *R*_P_ = *I*_ph_/*P*_ch_, the ratio of *I*_ph_, and illumination power over channel *P*_ch_ increased up to a factor of 3 (2.5 ± 0.8 averaged over all measured wavelengths) at ε = 0.9%. Such enhancement is seen and investigated in more detail with a second photocurrent spectroscopy setup, as described in the following paragraph. Device photoresponsivity is negligible for light wavelengths larger than 680 nm (cut-off value), which signifies that photons do not have enough energy to excite electrons from the valence to the conduction band, i.e., electrons cannot surpass the 1.8 eV optical bandgap of monolayer MoS_2_.^47^ At lower wavelengths, higher-energy electrons are excited, and the photocurrent is generated. The excitation increases for higher photon energies, which can be seen in the increase of the photocurrent and subsequently photoresponsivity down to the 400 nm excitation wavelength.^48^

For a more detailed inspection of the photoresponsivity behavior under strain, a second setup was used in the photon energy range corresponding to expected exciton energies (580–700 nm) with the ability to change the illumination wavelength in 5 nm steps continuously, as described in Methods. Figure 3d shows the plot of *I*_ph_ at different amounts of ε for wavelengths from 585 to 695 nm. In that wavelength range, energies corresponding to the B (604 nm = 2.05 eV) and A (645 nm = 1.92 eV) excitons in MoS_2_ are found. Again, like in LED light spectroscopy shown in Figure 3c, with an increase in ε, photoresponsivity increases and the spectra red-shifts, in agreement with previous reports.^22,49^ Each spectrum was fitted with two Lorentzians to determine the amount of the redshift. The corresponding GFs calculated for a shift of the A and B excitons are GF_A_ = −51 ± 6 meV/% and GF_B_ = −29 ± 4 meV/%, as shown in Figure S4. These values agree with previous reports, which utilized the same techniques and similar setups, confirming adequate strain transfer during all measurements.^22,27^ It is visible that the device cutoff point increases to higher wavelengths with the application of ε due to the redshift of spectra. Our results confirm that the MoS_2_ photoresponse can be enhanced by applying ε. Photocurrent and photoresponsivity increase by a factor of 3 at 0.9% of ε, and the device functionality is broadened to larger wavelengths; see Figure S3. Our device is dominated by a photogating mechanism that gets more pronounced by strain, which is determined by the longer response time at higher ε. The distance between atoms increases with ε, which, most likely, prolongs hole trapping in midgap states, occurring on grain boundaries and defects.^22,50,51^ At the same wavelength and applied voltage, the photocurrent increases for higher strain levels. This means that carrier mobility, which affects the time the electron needs to reach the drain electrode or trap the holes, increases. Because our response time increases with ε, the trapping of the holes is increased, which causes the photocurrent to rise. However, whether the number of possible trap states increases or the holes stay trapped for a longer time and what exact microscopic mechanism causes the increase in hole trapping are still unclear. Various studies noticed similar behavior of the photoresponse of strained MoS_2_ photodetectors but did not investigate the microscopic mechanism that could explain such a change on different strains.^22,52^ Previous reports show that in CVD-grown MoS_2_ monolayers, most trap states are located around grain boundaries.^51^ Notably, as we demonstrated in our previous work by Niehues et al.,^27^ the CVD-grown large-area MoS_2_ monolayer sheet is polycrystalline, with typical grain size from a few μm up to a few tens of μm,^53^ which implies that inside the investigated device channel, there are more than dozens of grain boundaries that can accumulate trap states. Grain boundary defects are accommodated mainly through the lattice distortions, and with applied strain, those distortions can be enhanced.^54^ In that way, it seems that with the application of strain, trap states in grain boundaries of large-scale MoS_2_ get modified, resulting in a more extended period of hole trapping, which in the end results in increased photo gain at higher levels of strain.

As shown in Table S1, in contrast to previous publications, our work is based on large-scale MoS_2_ monolayers (1 cm^2^ sheet area with device channel length *L* = 25 μm), where we have shown that with standard fabrication steps, one can utilize MoS_2_ sheets as strain-enhanced photodetectors and strain sensors. Although our device performances are at lower values than in previous reports, we have exploited methods that are friendly for industrial integration, which strongly supports further utilization of MoS_2_ monolayer sheets. Our reported values are smaller than those of state-of-the-art MoS_2_ devices mainly because of different intrinsic or transfer-induced defects and contaminations in our devices, which can be improved in future work with higher-quality CVD growth, smaller channel lengths, and optimized transfer protocols.

## 4 Conclusions

In this study, we showed that large-scale MoS_2_ monolayers are suitable for fabricating tactile and strain sensors which exhibit reproducible operation over a long period of time without applying any encapsulation, thus proving to be attractive for further MoS_2_ functionalization and novel utilities. Most importantly, by performing strain-dependent photocurrent spectroscopy of fabricated devices, we have unambiguously demonstrated that MoS_2_ photoresponsivity can be enhanced by a factor of 3 with an application of tensile strain less than 1%. The performances of our devices are at lower values than in previous reports, which most likely arise from intrinsic or transfer-induced defects and contaminations in the channel that can be minimised in further work and subsequently optimise device performances. Our work strongly supports the utilization of large-scale MoS_2_ monolayers for novel flexible and optoelectronic devices that are durable and whose operability can be enhanced by applying strain to their 2D material basis.

## Supporting Information Available

Schematic MoS_2_ transfer over the patterned PC sheet; AFM topographic images of MoS_2_ over a flat area of the PC sheet and over the channel; continuous measurement of the MoS_2_ sheet current as a function of time with cyclic exposure to light under 0, 0.3, and 0.9% of tensile strain; determined GF_P_ derived from resistance measurements up to 1% of strain and GF_A_ and GF_B_ derived from photocurrent spectroscopy under strain up to 0.9%; and comparison of device properties from our work with previous publications (PDF)
