# Supplementary material for: Strain-Enhanced Large-Area Monolayer MoS2 Photodetectors
Source: ACS Appl Mater Interfaces. 2024 Mar 19;16(12):15596–604. doi: 10.1021/acsami.4c00458 (PMC10982932; doi:10.1021/acsami.4c00458)
Supplement: Supplementary file 1 — am4c00458_si_001.pdf [file am4c00458_si_001.pdf]

Supporting Information:

Strain-enhanced large-area monolayer MoS_2 photodetectors

Borna Radatović [1,2], Onur Çakıroğlu [2], Valentino Jadriško [1,3], Riccardo Frisenda [4], Ana Senkić[1], Nataša Vujičić [1], Marko Kralj [1], Marin Petrović [1], Andres Castellanos-Gomez [2*]*

[1] Center for Advanced Laser Techniques, Institute of Physics, Bijenička 46, 10000 Zagreb, Croatia

[2] Materials Science Factory Instituto de Ciencia de Materiales de Madrid (ICMM-CSIC), 28049 Madrid, Spain

[3] Politecnico di Milano, Physics Department, 20133 Milan, Italy

[4] Physics Department, Sapienza University of Rome, 00185 Rome, Italy

E-mail: bradatovic@ifs.hr; andres.castellanos@csic.es

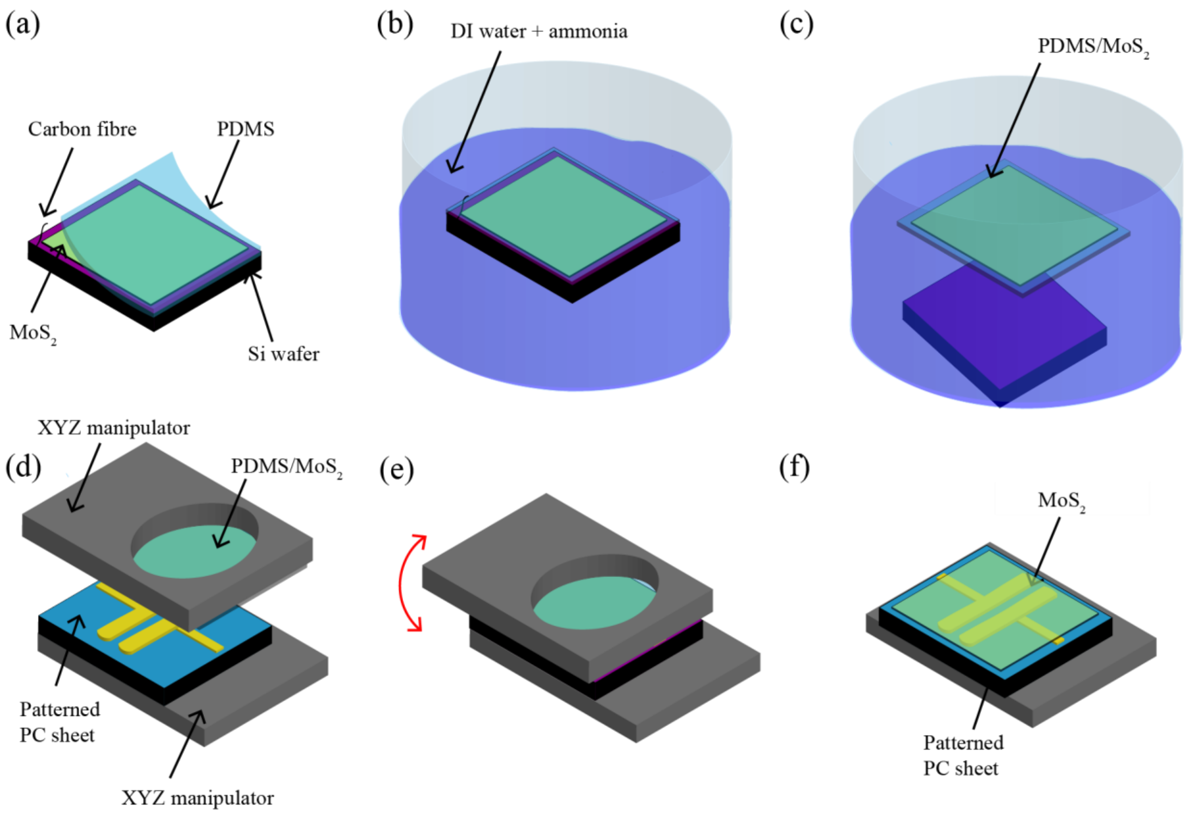

Figure S1. Schematic MoS_2 transfer over patterned PC sheet. (a) PDMS layer is placed on top of MoS_2 grown on Si wafer. (b) The sample is placed to float on top of DI water and Ammonia solution. (c) PDMS/MoS_2 is separated from the wafer that sinks to the bottom of the solution in a few minutes. (d) PDMS/MoS_2 is positioned with an XYZ manipulator over the targeted area on the patterned PC sheet substrate and connected and pressed slightly to ensure uniform adhesion with the substrate. (e) PDMS is slowly separated with a z-axis manipulator, leaving MoS_2 on a new substrate. (f) MoS_2 is transferred onto the patterned PC sheet.

The PDMS stamp, bought from the "Gel Pak" company, was cut into a rectangle and placed on CVD-grown MoS_2 on a Si/SiO_2 wafer that was purchased from the "2D Semiconductors" company, as illustrated in Figure S1(a). To increase the speed of the next step, PDMS is left without contact on one of the edges of the Si wafer, which can be ensured by placing a carbon fibre or any similar object on the edge of the wafer before placing PDMS on top. Figure S1(b)

shows an illustration of the pick-up step during which a PDMS / MoS_2 / Si wafer is placed on top of the solution of 100 ml DI water with a few drops of ammonia (100 µL), which is gently stirred in start to ensure the flow of the solution. The solution slowly intercalates between PDMS and Si wafer, separating 2D materials from the Si wafer. After the complete separation in a few minutes, Si wafer sinks into the solution, and PDMS with MoS_2 is left floating on top of the solution, as shown in Figure S1(c). Ammonia is known to dissolve alkali-like metals and is often used during the intercalation of 2D materials.[1] It is added into DI water to improve the removal of any ions that could dope 2D materials afterwards, but pure DI water could also be used for this step.[2] Figure S1(d) shows the step during which PDMS with MoS_2 is placed onto the patterned PC sheet. A pair of Ti/Au electrodes were previously patterned by e-beam evaporation and shadow mask lithography using a metal stencil from the company "Ossila". During placing, the PDMS stamp is gently pressed with an ear stick or simply by hand to ensure adequate adhesion over the whole substrate. This step is monitored by an optical microscope or camera and controlled with a set of micromanipulators with which one can precisely control the position and separation of PDMS and substrate.[3] To ensure MoS_2 has higher adhesion with the substrate, PDMS is slowly separated in small steps with a z-axis manipulator, as shown in Figure S1(e). After the complete separation of PDMS, MoS_2 is left on top of the new substrate, as shown in Figure S1(f). The front of the adhesion is monitored via an optical microscope to increase separation speed in the areas of no interest and reduce it in the areas where MoS_2 is targeted for transfer.

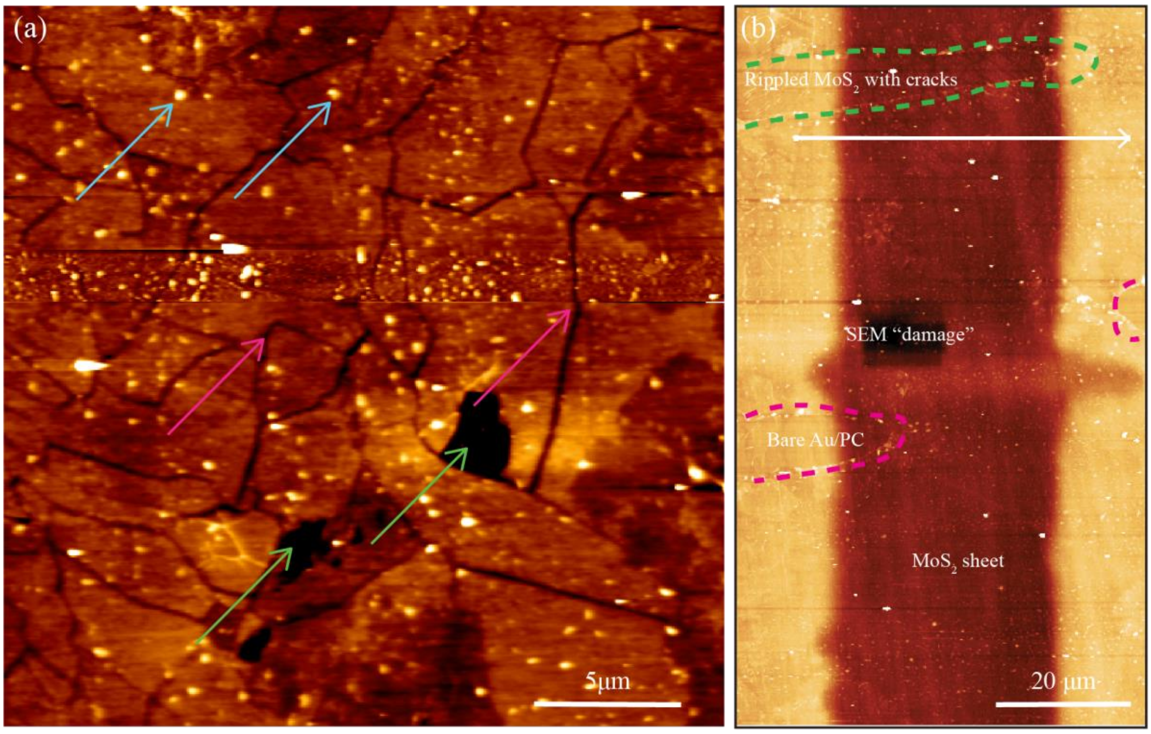

Figure S2. AFM topographfs of MoS_2. (a) Image taken over a flat area of the PC sheet with indicated contaminations, cracks and holes by cyan, magenta and green arrows, respectively. (b) Image taken over the channel, with visible SEM-induced damage due to PC charging from the e-beam. Pink dashed lines indicate a bare Au/PC without MoS_2 due to blistering around contaminations, while the green dashed line indicates an area where MoS_2 rippled and cracked due to the same contaminations.

Figure S2(a) shows the AFM topography of the MoS_2 monolayer sheet after transfer on PC patterned with Au electrodes in the region outside Au electrodes. It is important to emphasize that our device channel was around 25 μm, which prevented avoiding indicated post-transfer contaminations and cracks that can lead to current reduction and lower reported GF_P. The majority of the monolayer sheet was uniformly transferred without any significant damage, as shown in Figure S2(b). Contaminations, most likely PDMS residue from the transfer, scarcely cover the MoS_2 surface. However, certain contamination arises from the substrate. In some

regions, MoS_2 was not transferred, indicated by a pink dashed line, while in some areas, cracks were induced, indicated by a green dashed line. Both types of regions are surrounded by a ring of contaminations, whose morphology most likely causes local blistering of MoS_2, resulting in cracks or inadequate transfer.

Additionally, more minor cracks in the channel could be present, which are not distinguishable in Figure S2(b) due to the high RMS_q roughness ≈ of 8 nm inside the channel.[4,5] It is important to note that these defects can be further minimized with annealing treatments or previously reduced by optimized transfer protocols such as heat application or general usage of the glove box during the transfer. Most importantly, we have used various PDMS separation front speeds, as described in Figure S1(e), while a slower and constant speed would result in lower transfer-induced defects and contaminations.

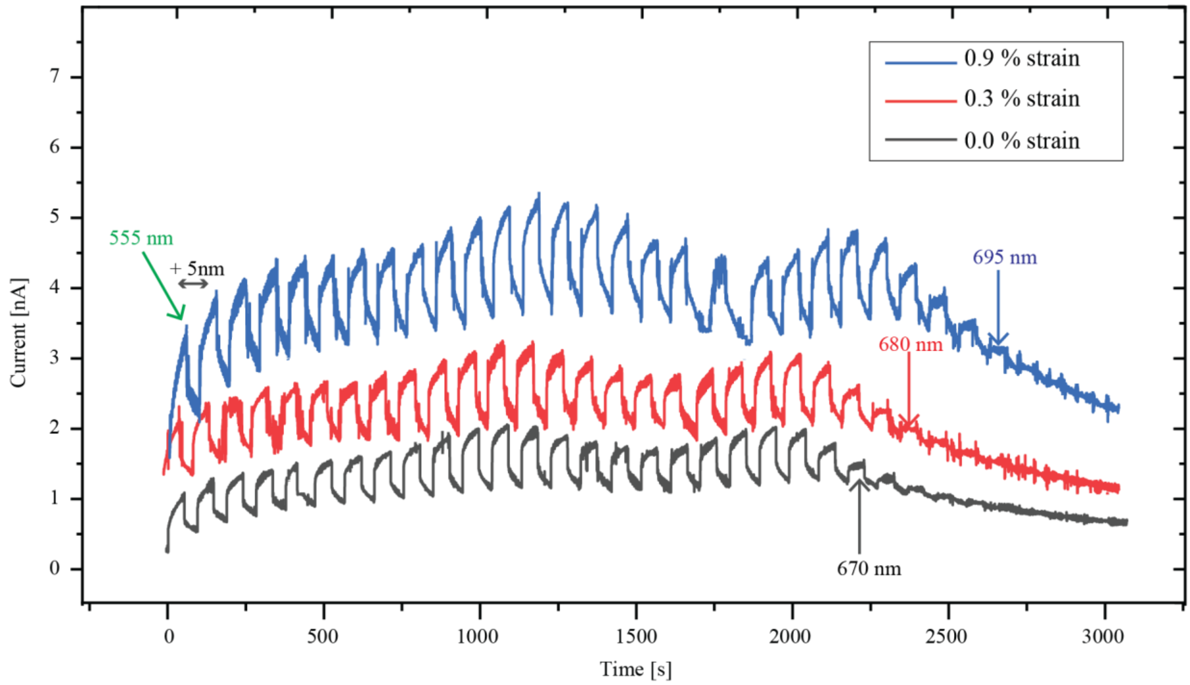

Figure S3. Continuous measurement of MoS_2 sheet current as a function of time with cyclic exposure to light, where each subsequent peak corresponds to illumination by light with a 5 nm

higher wavelength than the previous peak. Measurements for 0.0%, 0.3% and 0.9% of strain are shown, where the first wavelength was 555 nm, while cut-off wavelengths are marked correspondingly for each strain value.

Figure S3 shows photocurrent measurement by a continuous light source for 0.0%, 0.3%, and 0.9% of induced ε, with the first peak of each curve corresponding to 550 nm and each subsequent peak to 5nm increase of light source wavelength. Even on unprocessed data, it can be seen that the total current increases with an increase in strain. Due to persistent photoconductivity (PPC), the device's conductance was enhanced after the illumination was terminated and remained in a high-conductivity state during the whole measurement.[6] This delayed phenomenon is governed by the relaxation of the excess electrons in the conduction band to hole-trapped sites.[7] It can be seen that at higher amounts of the applied strain, the PPC effect is also increased, which results in the larger photo gain at higher ε.

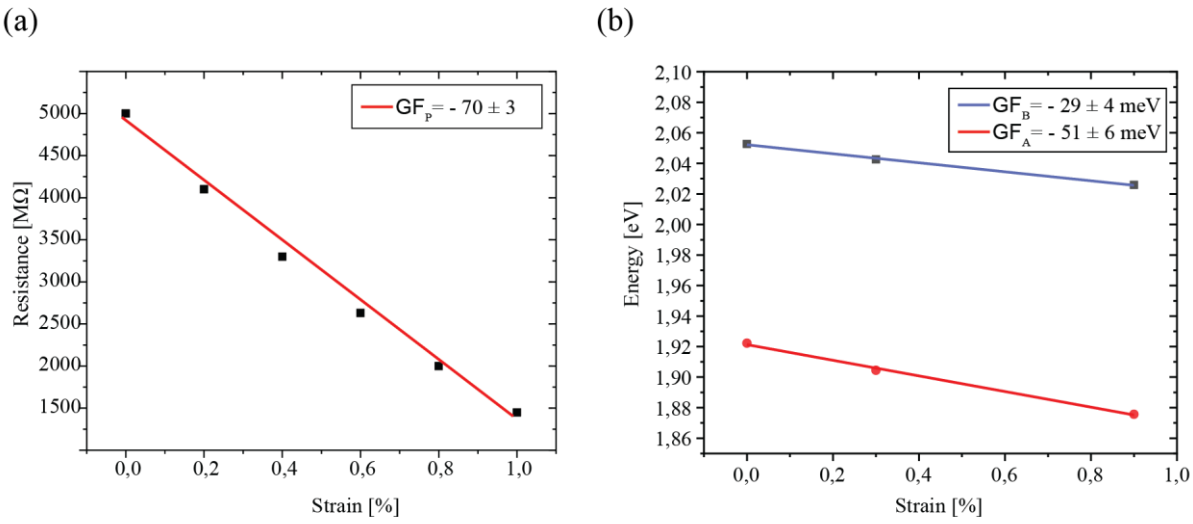

Figure S4. Determined GFs. (a) GF_P derived from resistance measurements up to 1% of strain. (b) GF_A and GF_B derived from photocurrent spectroscopy under strain up to 0.9%.

Figure S4 shows GFs derived from strain-dependent characterizations. GF_P was derived from strain-dependent resistance measurement illustrated in Figure 2(a), while GF_A and GF_B were derived from strain-dependent photocurrent spectroscopy shown in Figure 3(d).

Table S1 compares device properties from our work with prior publications on MoS_2-based flexible photodetectors and strain sensors. In contrast to earlier publications, devices in this work were based on a MoS_2 monolayer sheet with a lateral size of over 1 cm and a device channel width of 250 μm. Notably, most research on MoS_2 monolayers was conducted with exfoliated samples, while CVD monolayers were usually multilayers or chemically modified samples. Although we did not optimize our devices' performances (e.g. annealing, encapsulating, gating, smaller channel dimensions, higher applied voltage and laser power), we achieved performance comparable to prior reported values. It is important to note that we investigated both photodetectors and strain sensors, while previous publications mainly focused on a single type of device and the improvement of its properties.

Table S1. Comparison of device properties from our work with previous publications.

	Our work	Gant et al.[8]	Datye et al.[9]	Çakıroğlu et al. [10]	Li et al. [11]	Park et al. [12]	Zhu et al. [13]
MoS_2 type	CVD mono*	Exf** mono*	Exf** mono*	Exf** mono*	CVD mono*	Encap*** CVD multi****	V doped CVD mono*
Material size	1 cm	20 µm	10 µm	30 µm	30 µm	1 mm	100 µm
Strain [%]	0 ↔ 1	-0.8 ↔ 0,48	0 ↔ 0.7	0 ↔ 1	0 ↔ 1.4	-2 ↔ 2	-0.04 ↔ 0.04
GF_A [meV/%]	-51 ± 6	-94	-63 ± 10	-55 ± 4	NA	NA	NA
GF_P	70 ± 3	NA	140	102 ± 21	NA	72,5	140
V_{DS} [V]	1	5 ↔ 10	NA	NA	1 ↔ 5	NA	NA
I_{on}/I_{off}	2	3	NA	NA	5	NA	NA
Response time [s]	4 ↔ 8	80 x 10^{-3} ↔ 1.5	NA	150 x 10^{-3}	45 ↔ 115	NA	NA
Max. I_{ph} [nA]	0.1 ↔ 10	1 ↔ 15	NA	NA	370 ↔ 2 x 10^3	NA	NA
Max R [ma/W]	0.4↔ 5	10 ↔ 200 x 10^3	NA	40	100 ↔100 x 10^3	NA	NA

* monolayer

** exfoliated

*** encapsulated

**** multilayer

Supplementary references

(1) Rajapakse, M.; Karki, B.; Abu, U. O.; Pishgar, S.; Musa, M. R. K.; Riyadh, S. M. S.; Yu, M.; Sumanasekera, G.; Jasinski, J. B. Intercalation as a Versatile Tool for Fabrication, Property Tuning, and Phase Transitions in 2D Materials. *npj 2D Mater. Appl. 2021 51* **2021**, *5* (1), 1–21. https://doi.org/10.1038/s41699-021-00211-6.

(2) Hassanpour Amiri, M.; Heidler, J.; Hasnain, A.; Anwar, S.; Lu, H.; Müllen, K.; Asadi, K. Doping Free Transfer of Graphene Using Aqueous Ammonia Flow. *RSC Adv.* **2020**, *10* (2), 1127–1131. https://doi.org/10.1039/C9RA06738H.

(3) Castellanos-Gomez, A.; Buscema, M.; Molenaar, R.; Singh, V.; Janssen, L.; Van Der Zant, H. S. J.; Steele, G. A. Deterministic Transfer of Two-Dimensional Materials by All-Dry Viscoelastic Stamping. *2D Mater.* **2014**, *1* (1), 011002. https://doi.org/10.1088/2053-1583/1/1/011002.

(4) Lu, S.; Jia, H.; Gu -, Z.; Ma, L.; Wang, C.; Chu, Y.; -, al; Pan, M.; Yuan, H.; Cao -, Y.; Jain, A.; Bharadwaj, P.; Heeg, S.; Parzefall, M.; Taniguchi, T.; Watanabe, K.; Novotny, L. Minimizing Residues and Strain in 2D Materials Transferred from PDMS. *Nanotechnology* **2018**, *29* (26), 265203. https://doi.org/10.1088/1361-6528/AABD90.

(5) Fan, S.; Vu, Q. A.; Tran, M. D.; Adhikari, S.; Lee, Y. H. Transfer Assembly for Two-Dimensional van Der Waals Heterostructures. *2D Mater.* **2020**, *7* (2), 022005. https://doi.org/10.1088/2053-1583/AB7629.

(6) Wu, Y. C.; Liu, C. H.; Chen, S. Y.; Shih, F. Y.; Ho, P. H.; Chen, C. W.; Liang, C. Te; Wang, W. H. Extrinsic Origin of Persistent Photoconductivity in Monolayer MoS2 Field Effect Transistors. *Sci. Rep.* **2015**, *5* (1), 1–10. https://doi.org/10.1038/srep11472.

(7) Tran, M. D.; Kim, J. H.; Kim, H.; Doan, M. H.; Duong, D. L.; Lee, Y. H. Role of Hole Trap Sites in MoS2 for Inconsistency in Optical and Electrical Phenomena. *ACS Appl. Mater. Interfaces* **2018**, *10* (12), 10580–10586. https://doi.org/10.1021/acsami.8b00541.

(8) Gant, P.; Huang, P.; Pérez de Lara, D.; Guo, D.; Frisenda, R.; Castellanos-Gomez, A. A Strain Tunable Single-Layer MoS2 Photodetector. *Mater. Today* **2019**, *27*, 8–13. https://doi.org/10.1016/j.mattod.2019.04.019.

(9) Datye, I. M.; Daus, A.; Grady, R. W.; Brenner, K.; Vaziri, S.; Pop, E. Strain-Enhanced Mobility of Monolayer MoS2. *Nano Lett.* **2022**, *22* (20), 8052–8059. https://doi.org/10.1021/acs.nanolett.2c01707.

(10) Çakıroğlu, O.; Island, J. O.; Xie, Y.; Frisenda, R.; Castellanos-Gomez, A. An Automated System for Strain Engineering and Straintronics of 2D Materials. *Adv. Mater. Technol.* **2023**, *8* (1), 2201091. https://doi.org/10.1002/admt.202201091.

(11) Li, F.; Shen, T.; Xu, L.; Hu, C.; Qi, J. Strain Improving the Performance of a Flexible Monolayer MoS2 Photodetector. *Adv. Electron. Mater.* **2019**, *5* (12). https://doi.org/10.1002/AELM.201900803.

(12) Park, M.; Park, Y. J.; Chen, X.; Park, Y. K.; Kim, M. S.; Ahn, J. H. MoS2-Based Tactile Sensor for Electronic Skin Applications. *Adv. Mater.* **2016**, *28* (13), 2556–2562. https://doi.org/10.1002/adma.201505124.

(13) Zhu, M.; Li, J.; Inomata, N.; Toda, M.; Ono, T. Vanadium-Doped Molybdenum Disulfide Film-Based Strain Sensors with High Gauge Factor. *Appl. Phys. Express* **2019**, *12* (1), 015003. https://doi.org/10.7567/1882-0786/AAF5C4.
